# Unlocking Real-Time Data Access in Diabetes Management: Toward an Interoperability Model

**DOI:** 10.1177/19322968251327602

**Published:** 2025-03-28

**Authors:** Pietro Randine, Miriam Kopperstad Wolff, Matthias Pocs, Ian R. O. Connell, Joseph A. Cafazzo, Eirik Årsand

**Affiliations:** 1 Department of Computer Science, Faculty of Science and Technology, UiT The Arctic University of Norway, Tromsø, Norway; 2 Department of ICT and Natural Sciences, Faculty of Natural Sciences, Norwegian University of Science and Technology, Trondheim, Norway; 3 STELAR Security Technology Law Research, Hamburg, Germany; 4 Department of Medical Biophysics, Temerty Faculty of Medicine, University of Toronto, Toronto, ON, Canada; 5 Biomedical Engineering, University Health Network, Toronto, ON, Canada; 6 Institute of Health Policy, Management and Evaluation, Dalla Lana School of Public Health, University of Toronto, Toronto, ON, Canada

**Keywords:** diabetes management, interoperability, openness, real-time data access, FHIR (Fast Healthcare Interoperability Resources)

## Abstract

**Background:**

In today’s data-driven era, openness promotes transparency and accessibility, particularly in health initiatives like the European Health Data Space. Diabetes management relies on real-time data from medical devices, such as continuous glucose monitors (CGMs), insulin pumps, and hybrid closed-loop systems. These devices provide critical insights for treatment adjustments, making real-time data access essential.

**Methods:**

This article explores real-time data access for third-party applications, focusing on primary (treatment) and secondary (research) use. We examine how application programming interfaces (APIs) enable secure data retrieval and assess the impact of terms of service and copyright law on patient-driven innovation in open-source communities. Our research evaluates diabetes medical devices and software solutions in Norway, assessing their real-time data access and API functionalities. In addition, we analyze legal frameworks governing these technologies, focusing on challenges faced by open-source solutions. Based on our findings, we propose an interoperability model to improve data accessibility while ensuring security and transparency.

**Results:**

Findings reveal seven diabetes devices and nine regulated software solutions, with only one offering a publicly accessible API. This emphasizes a significant gap in real-time data access. Comparisons between vendor-specific and open-source software expose interoperability and accessibility challenges. While Do-It-Yourself (DIY) solutions foster innovation, they face technical and legal barriers.

**Conclusion:**

Real-time diabetes management presents security, transparency, and access challenges. Regulatory decisions are needed to implement an interoperability model. The lack of real-time data access highlights the necessity of publicly accessible APIs that prioritize transparency, accessibility, and patient-driven innovation—marking a shift from today’s constrained diabetes management landscape.

## Introduction

The concept of “openness” in the context of health data, software, and research is increasingly prominent, especially within the European Union (EU) and European economic area (EEA).^1^ This movement, characterized by transparency, participatory engagement, and data sharing,^2^ is exemplified by the European Health Data Space, which seeks to empower individuals and organizations through accessible and reusable health data.^3^ Complementing this trend is the General Data Protection Regulation (GDPR), which advocates for the rights of European citizens in data processing.^4^ Application programming interfaces (APIs) play a critical role in facilitating technical data access^5^ and promoting interoperability,^6^ aligning with the GDPR’s core principles. These user-friendly APIs empower developers to meet their data needs and usage objectives, fostering data access and GDPR compliance. Consequently, APIs unlock possibilities for Big Data applications,^7^ making it easier for different health care and research systems to work together.^8^

In the intricate and competitive field of diabetes management, “openness” presents unique challenges, especially concerning real-time data access,^9^ a topic we explore further in this article.

### Diabetes Management and Data Sharing

Diabetes is a common chronic disease managed with various devices, including blood glucose meters, blood ketone meters, continuous glucose monitors (CGMs), and insulin pumps. Whether stand-alone or connected, these tools often integrate with mobile apps for glucose tracking, physical activity, pregnancy management, and other health aspects, enhancing diabetes care.^10-13^

Managing a long-term chronic condition like diabetes requires ongoing monitoring, personalized treatment, and regular health care consultations.^14^ In addition to patients and health care providers (HCPs), other important contributors, such as informal caregivers (like family members and next of kin), play a crucial role, especially in the care of children. This highlights the need for real-time data sharing.^15,16^ Having access to accurate and up-to-date health information allows caregivers to offer better support.

Given these factors, it is more important than ever to establish effective and accessible data-sharing mechanisms for diabetes management. Strengthening interoperability between systems like electronic health record (EHR) and enabling secure, real-time data exchange among all stakeholders—including patients, caregivers, health care professionals, researchers, and developers—is essential. In addition, these elements play a vital role in driving innovation through research activities.^17^

### Innovation From Do-It-Yourself Communities and Off-Label Use

Diabetes management has significantly evolved with the Do-It-Yourself (DIY) movement, known under several hashtags on social media, such as #WeAreNotWaiting. Since its beginning in the United States in 2013, this movement has globally advanced diabetes technologies, particularly in open-source hybrid closed-loop systems and improved access to CGM data.^18-20^ Hybrid closed-loop systems were commercialized in 2016 but faced delayed accessibility in Norway and other countries, leading to reliance on DIY solutions.^21,22^ A similar situation is occurring in the 2020s with automated insulin delivery (AID) systems, reflecting hybrid closed-loop systems‘ initial challenges.^23^

Do-It-Yourself solutions have proven to be highly beneficial for individuals who strongly emphasize improving their quality of life. They offer convenient insulin management via smartphones, smartwatches, and other remote control features, which is particularly helpful for parents managing their children’s diabetes.^23^ These solutions have cultivated a strong online community where users exchange information and seek support.^24,25^ In some cases, they also coordinate activities, such as reverse-engineering original software or modifying vendor applications to gain more control over their medical devices.

Notable initiatives within the DIY movement in diabetes include OpenAPS^26^ (Open Artificial Pancreas System), Loop,^27^ and Nightscout^28^ (CGM in the cloud).

### Terms of Services, Copyright, and Cybersecurity

The use of diabetes medical devices in the EEA is governed by legal frameworks, including GDPR-compliant privacy policies and terms of service (ToS) documents^29^—which often cover intellectual property rights and copyright.

In the context of open-source communities using medical devices off-label, conflicts with manufacturer ToS and copyright agreements can arise. However, exceptions exist under copyright laws. Directive 2009/24/EC in the EEA allows limited use of copyrighted material for specific purposes like achieving interoperability.^30^ The implementation of this Directive varies among countries in the EEA. For example, Norway incorporates it through the Intellectual Property Act,^31^ while Germany does so through the Copyright Act.^32^ Despite these different approaches, both countries adhere to the same conditions as outlined in Table 1.

**Table 1.** Conditions to Copyright Exception Directive 2009/24/EC in Norway and Germany.

Condition for decompilation in Norway and Germany
1. The action is performed by a user who holds a valid license
2. The necessary information for achieving interoperability has not been provided to the user
3. Only the portions of the software that are essential for achieving interoperability can be manipulated

Cybersecurity is another crucial aspect, with the EU adopting, at the end of 2024, a new law on the European Cyber Resilience Act^33,34^ and the US Food and Drug Administration (FDA) issuing medical device security recommendations.^35^ Regulatory bodies are especially vigilant in diabetes, warning about using CGM devices in unauthorized diabetes management solutions.^36^ In response, manufacturers are proactively enhancing data security and safeguarding patient information, as they are responsible for ensuring software security in the products they release to the public.^33,35^

## Methods

This study explores real-time data access from diabetes medical devices approved in Norway, focusing on primary (treatment) and secondary (research) use cases. Central to our investigation is the role of APIs in facilitating secure and efficient data transfer.

We also investigate the feasibility of accessing the data using third-party software for research (secondary use) and treatment (primary use) and exclusively address real-time data. Data collection via the regulated software solution received ethical approval from the Norwegian Agency for Shared Services in Education and Research (SIKT N 671274).

We define “real-time” data in the context of diabetes as the capability to access and process data instantly or with minimal delay (maximum five minutes after registration), ensuring a timely and accurate reflection of the device’s status. The study concentrates on remote data access, excluding software solutions reliant on Bluetooth and Near-Field Communication (NFC) technologies.

### Objectives and Research Questions

To provide a clear and structured overview of our study, we have summarized the main objectives and associated research questions in Table 2.

**Table 2.** Objectives and Research Questions.

Objective	Research questions
Identify available diabetes medical devices in Norway and assess their real-time data access capabilities	**Research Question 1:** Real-time data from diabetes medical devices be accessed using regulated software solutions via publicly accessible APIs? **Research Question 2:** Is there any working off-label alternative available for real-time data access?
Evaluate the feasibility of using identified solutions for treatment (primary use) and research purposes (secondary use)	**Research Question 3:** Technologies are utilized for accessing real-time data from diabetes medical devices in third-party applications? **Research Question 4:** What are the key considerations within the terms of service governing the access to real-time data from diabetes medical devices in third-party applications?
Develop an interoperability model for data sharing and real-time access within third-party applications	Based on insights from RQ1 to RQ4

### Study Structure

**Figure 1.**
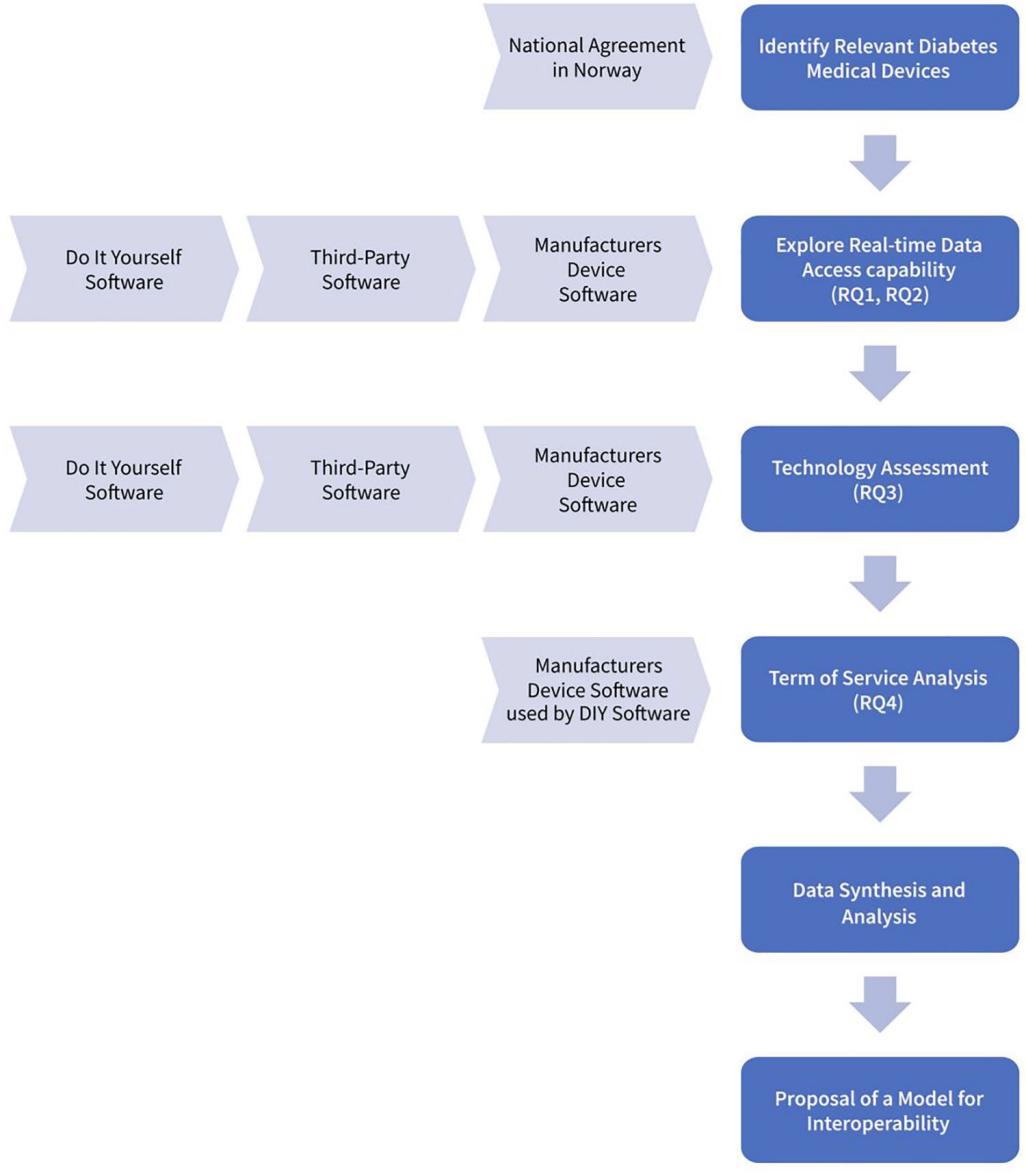
Study steps—visual representation.

A visual representation of the sequential steps involved in our study is presented in Figure 1. Each step contributes to the overarching goal of establishing better interoperability in diabetes data. These stages include identifying relevant diabetes medical devices and exploring third-party applications, including assessing the technology landscape, analyzing ToS, synthesizing data, and ultimately proposing a transformative interoperability model in diabetes.

## Results

### Medical Devices Available in Norway and Associated Regulated Software

Table 3 presents the available medical devices in Norway at the beginning of 2024^37^ and corresponding regulated software solutions capable of real-time data collection, reflecting the European context overall. In addition, we have reported cases where publicly accessible APIs are available for direct interaction with the data collected by the medical devices or associated software solutions.

**Table 3.** Medical Devices Available in Norway and Associated Regulated Software Able to Provide Real-Time Diabetes Data.

Medical equipment (*n* = 7)	Type	Regulated software that processes data (*n* = 9)	Publicly accessible APIs.
MiniMed 780G + Guardian Connect G4	Insulin pump and CGM—hybrid closed-loop system	CareLink Connect, MiniMed Mobile	No
Tandem t: slim X2™ Insulin pump Control-IQ + DXCM1 G6	Insulin pump and CGM—hybrid closed-loop system	Glooko	No
Omnipod DASH	Insulin patch pump	Not Available in Norway	No
Accu Check Solo	Insulin patch pump	RocheDiabetes Care Platform	No^38^
Freestyle Libre 2	CGM	LibreView Data Management System, LibreLink Up, FreeStyle Apps for Libre 2 and Libre 3	No
Freestyle Libre 3	CGM		
Dexcom G6	CGM	Dexcom G6 mmol/L DXCM1, Glooko	Yes^39^

Table 3 does not include the Eversense E3 sensor because it was scheduled for removal in 2024, as confirmed by national authorities. In addition, the Omnipod cloud service, which includes cloud functionality for the Omnipod DASH, is unavailable in Norway. Therefore, data transfer with the Omnipod DASH system required a wired connection through Glooko, which was excluded since it does not provide real-time data. Finally, while the RocheDiabetes Care Platform has a publicly documented API,^38^ it is not accessible to the public.

### DIY Software and Off-Label Use

To access real-time data from diabetes medical devices, we searched for publicly available repositories and explored DIY alternatives. We identified mobile applications: Loop,^27^ AndroidAPS^40^ and OpenAPS.^41^ These systems can operate with either iPhone or Android devices, although they may require additional hardware to establish connections.^42^ It is crucial to emphasize that while these solutions enable real-time data access, they also control insulin delivery. It means they offer read-only data functionalities and allow direct input to the insulin pump, thereby providing control over the treatment process.

We also identified two repositories designed to interface specifically with Libre 2 and Libre 3 sensors,^43^ and Dexcom G6.^44^

### Technological Assessment of Regulated and DIY Software

**Figure 2.**
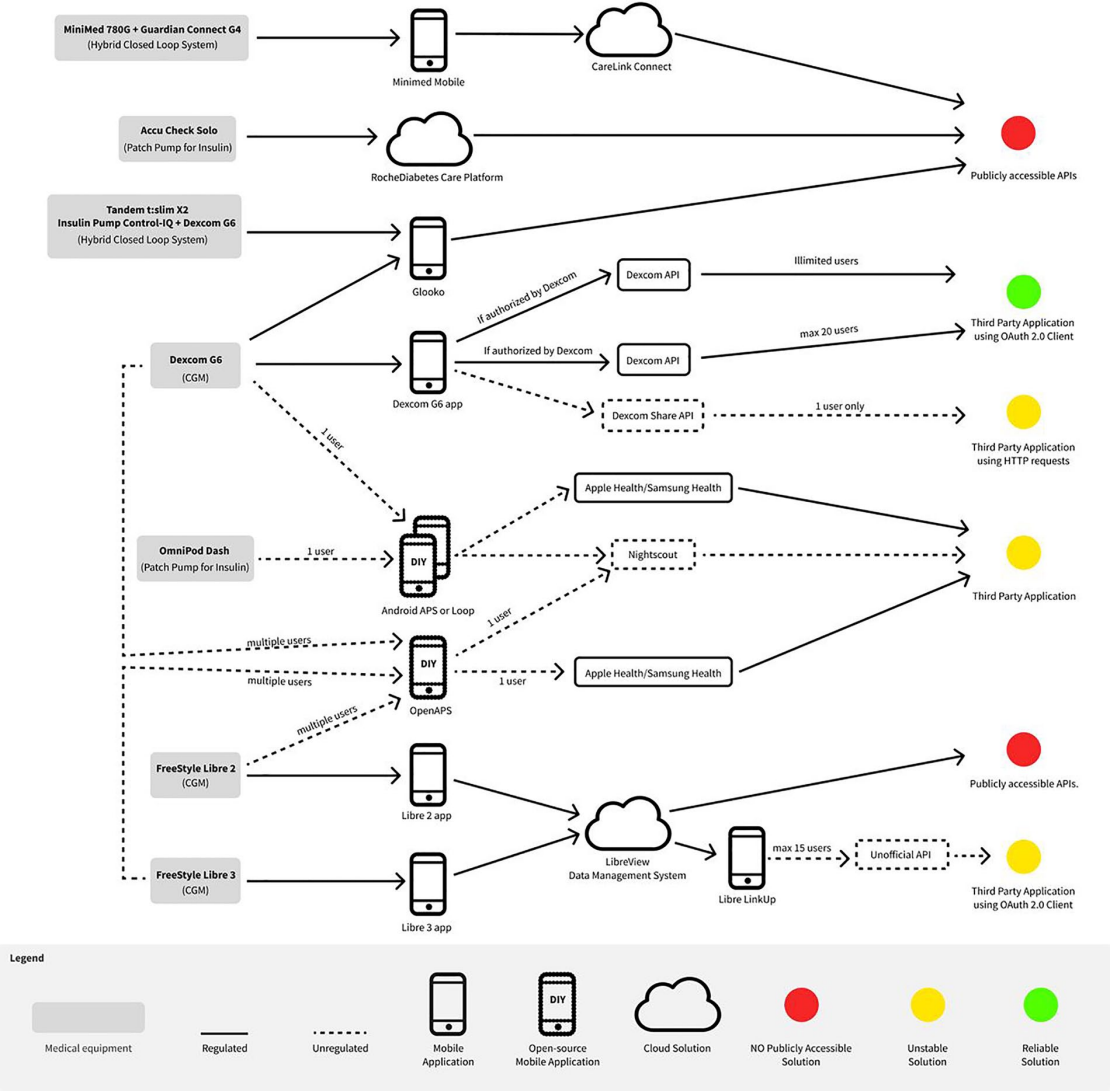
Technological assessment overview of regulated and DIY software.

The technological assessment for all the software solutions identified is illustrated in Figure 2. This assessment follows two distinct paths: the regulated and the unregulated path. We also consider cardinality, which indicates how many users can connect via third-party applications for real-time data access, serving primary (eg, treatment) or secondary (eg, research) purposes. Some of the identified solutions need supplemental software for data retrieval.

Regarding medical equipment, the DIY software is designed to interface with the Libre 2 and Libre 3 sensors using the LibreLink Up app^45^ or the Dexcom G6 mmol/L DXCM1 app.^44^ The Omnipod DASH may connect with Loop and AndroidAPS systems, while newer Medtronic devices like MiniMed 780G are unavailable for open-source software. Open-source software enables data sharing with other software like Apple Health, Samsung Galaxy, and Nightscout, facilitating real-time data collection. Table 4 lists the limitations of the seven identified DIY software solutions.

**Table 4.** Software Solutions That Enable Real-Time Access to Data Sharing and Their Limitations.

Solution identified (software category)	Limitation for a third-party application	Devices
OpenAPS (DIY software)	Additional software involved (Nightscout, Samsung Health, etc) Taking control over the insulin delivery in the pump	Dexcom G6, Freestyle Libre 2 and Libre 3
Android APS (DIY software)	1-1 user cardinality Additional software involved (Nightscout, Samsung Health, etc) Taking control over the insulin delivery in the pump	Omnipod DASH, Dexcom G6, Freestyle Libre 2 and Libre 3
Loop (DIY software)		
Dexcom Official (manufacturers’ device software)	None	Dexcom G6
Dexcom Authorized (manufacturers’ device software)	20 users with 30-minute delay (not real-time)	
Dexcom DIY solution (DIY software)	1-1 user cardinality requiring plain username and password credentials for access	
Libre (DIY software)	Limit to 15 users with one LibreLink Up account Non-functional if 2FA (two-factor authentication) is enabled	Libre 2 and Libre 3

### ToS Analysis

The solutions identified in Table 4 and visualized in Figure 2 are either based on or require the use of copyrighted software. This is the case for Freestyle Libre 2 and Freestyle Libre 3, requiring the official LibreLink Up app. In the ToS, among the statements of “Prohibited Uses,” users agree not to: “interfere with or disrupt the LibreView Data Management System (including accessing the LibreView Data Management System through any automated means, such as scripts or web crawlers),” and “reverse engineer, decompile, disassemble, decode, create derivative works of, gain access to the source code, or modify the LibreView Data Management System except and then solely to the extent permitted under applicable law.”^46^ The term “applicable law” is a generic term that encompasses all laws relevant to this agreement.

Similarly, Dexcom applies a similar restriction: “Reverse engineer or derive the source code for any DexCom Product, DexCom Service, or Software App not provided to you in source code form, except to the extent such restriction is expressly prohibited by applicable law.”^47^ The same principles apply to the Omnipod DASH.^48^

Among the different “applicable laws,” the implementation of the Directive 2009/24/EC is one of these, and it provides exceptions for achieving interoperability. Even with such exceptions, direct use of these DIY software would not be feasible for large-scale use, even without technical limitations. Moreover, even if it were legally possible to employ these solutions, the existing technical constraints, as outlined in Table 4, would render them impractical for large-scale use and, in most cases, limited to 15 users or limited security measures.

### Interoperability Model for Diabetes Data

The findings highlight a significant gap in real-time data access for diabetes management, as evident in the regulated (Table 3) and DIY solutions (Table 4). To address this issue, we propose an interoperability model tailored to enhance diabetes data accessibility.

Given the unavailability of real-time data from any hybrid closed-loop system, we investigated data generated by the hybrid closed-loop system, using the MiniMed 780G as an illustrative example. This system offers an option to produce a proprietary CSV file, which generates historical data in 32 columns and additional metadata.^49^ We also developed a code that analyses the patient-generated data.^50^ The data volume can be significant, typically comprising thousands of CGM glucose readings, hundreds of manual glucose measurements and carbohydrate estimations, and approximately a thousand insulin injections per month—distributed across closed-loop auto-correction and patient input.

A typical need is the post-processing of data using internal algorithms, including glucose measurements, insulin data, carbohydrate information and related metadata relevant to both primary and secondary use.

### A Set of Standards to Ease Diabetes Data Transfer

In diabetes data management, there are established standards and practices designed to ensure interoperability. We have technically demonstrated this interoperability model in our work, detailed in Randine.^51^ This implementation specifically illustrates how we realized real-time data access from Libre medical devices. We achieved this by adapting the DIY repository^45^ to illustrate its application in both primary and secondary use cases. This demonstration^51^ provides a practical example of how interoperability can be achieved in diabetes data management, a more efficient data flow between different systems and platforms.

The proposal aligns with international best practices, and it is based on recommendations for the European Electronic Health Record exchange format,^52^ other standardization projects focusing on diabetes data^53,54^ and the RocheDiabetes Care Platform implementation.^38^

**Figure 3.**
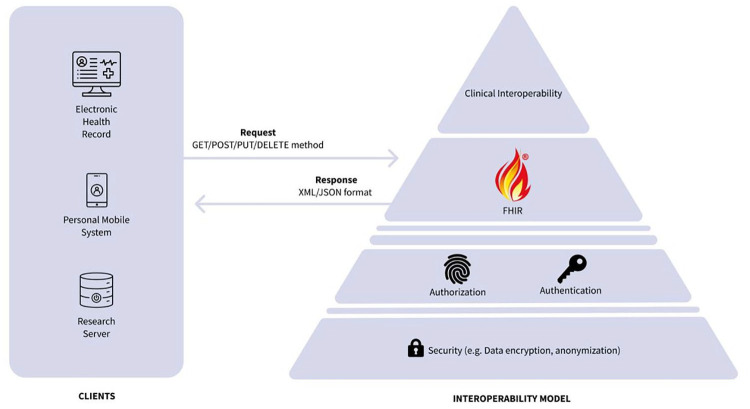
Proposal for a set of standards that will enable diabetes data sharing between end-users, equipment, vendors, researchers, and health care services.

The technological proposal is summarized in Figure 3. To promote interoperability and the reuse of diabetes health care data, the proposal advocates for adopting the widely accepted and user-friendly REST API architecture,^55^ with requests and responses. Furthermore, it recommends implementing authorization (eg, OAuth 2.0) and authentication (eg, OpenID Connect) protocols to enhance security and control data access. In addition, the proposal underscores the importance of two-factor authentication (2FA), recognized as one of the most effective security measures,^56^ to further fortify patient data protection.

For integration with EHR systems and health care settings, the proposal embraces the Fast Healthcare Interoperability Resources (FHIR) standard.^57^ FHIR is designed to ensure efficient data exchange across diverse health care platforms.^58^ Within the FHIR framework, specific resources are tailored to represent critical data elements effectively. The proposal includes the use of clinical terminology (eg, SNOMED CT, LOINC) codes to ensure precise clinical interoperability, aligning with the EU’s intention for cross-border data exchange.^59^

## Discussion

The interoperability challenges and barriers in diabetes health care are widely recognized.^60^ Our study, set in the Norwegian context, identified seven diabetes medical devices and nine regulated software solutions, with the Dexcom G6 CGM device being a notable exception in offering publicly accessible APIs (Table 3). Meanwhile, the RocheDiabetes Care Platform has a publicly FHIR-based documented API that is not directly accessible to the public.^38^ The limited availability of official real-time data access solutions prompted us to explore seven DIY software alternatives (Table 4). These alternatives revealed technical and practical limitations (Table 4), underscoring the impracticality of regulated and off-label medical device use for real-time data access in diabetes, particularly in hybrid closed-loop systems.

The crucial insight from our research is the availability of the required technology for effective data access, but the lack of cohesive governance, policy, and security measures in diabetes data processing complicates its use and reuse. To counter these challenges, we introduce an interoperability model for diabetes (Figure 3) aiming for a transformative shift in treatment and health care practices by leveraging existing standards.

### Why is a Model for Interoperability Needed?

The data fragmentation evident in diabetes management highlights the urgent need for a regulated interoperability model. While some APIs, such as Dexcom, provide valuable solutions,^39^ they represent a rare exception in a landscape where the norm is limited real-time data access for crucial activities like treatment and research. This situation contrasts with other sectors where APIs promote interoperability and innovation.^61^

Current software, primarily controlled by device manufacturers, often restricts data access. Typically, these software tools are designed not as comprehensive EHR systems but as analytical and reporting tools for HCPs, leading to a reliance on manual data entry into EHRs.^62^ This manual process increases the workload of HCPs, introduces potential errors and inconsistencies in patient records^63^ and elevates the risk of medical errors.^64^ Furthermore, given the time constraints faced by HCPs,^65^ the need for a standardized and accessible interoperability framework in diabetes care becomes imperative, allowing HCPs to prioritize patient treatment and consultation over the burden of data access.

### Enhancing Diabetes Data Access for Citizens

Patients and informal caregivers often encounter difficulties downloading diabetes data.^66,67^ Although device manufacturers provide machine-readable CSV files to comply with the GDPR, our study found that none of the hybrid closed-loop systems in Norway enable real-time data collection through public APIs or DIY solutions. The complexity and incomplete documentation of these data^49^ may potentially conflict with the GDPR’s Right to Data Portability,^4^ making it challenging for individuals without advanced technical skills to effectively reuse their data, as also observed in our testing.^50^ This raises an important question: Does giving patients a CSV file—often challenging to understand—truly allow them to “own” their data? While the GDPR grants individuals rights over their health information, the absence of standardized and user-friendly formats limits practical accessibility and control.

The lack of a standardized data exchange format, resulting in proprietary formats, makes it challenging to promote openness and data reuse by the European Health Data Space.^3^ To address these challenges, we propose implementing a FHIR-based RESTful architecture with publicly accessible APIs (Figure 3) to facilitate easier data exchange among EHRs and research initiatives. In addition, simplifying data representation would enhance patients’ understanding of these data.

### Navigating the Complex Landscape of DIY Diabetes Management Solutions

Do-It-Yourself users prioritize functionality over regulatory concerns,^68^ with limited technical skills and the need for technology maintenance being their primary apprehension.^69^ Our study findings validate this concern due to identified technical complexities (see Table 4, Figure 2). Unlike CE (Conformité Européene)-marked and Medical Device Regulation (MDR)-approved systems, which adhere to security regulations, DIY solutions may provide valid technical functionality. Still, they could raise concerns regarding data privacy, security, and the accountability of HCPs.^23^

Legal frameworks like Directive 2009/24/EC, as implemented in Norway and Germany,^31,32^ set conditions for software use, focusing on interoperability rather than new functionalities or research applications. The technical constraints of DIY solutions (Table 4) make them impractical for large-scale projects and health care institutions. Security concerns, such as the lack of 2FA and reliance on plain passwords, make this solution unsuitable for future use.^33,35^

Despite these challenges, patients and caregivers may still opt for DIY solutions due to their valued functionality and good community support. However, HCPs cannot formally recommend these unregulated solutions,^70^ and users might risk legal liability under the Medical Devices Act and Product Liability Act.^33^ Manufacturers are aware of these solutions, which are often publicly accessible and may expose software vulnerabilities, as we have also tested and observed in our testing.^51^

### Limitations

Our study addresses the key research questions (RQ1-RQ4) and offers important insights, and it is geographically limited to Norway. However, these findings may still offer relevance to the broader EEA/EU context and outside Europe, as EEA countries do not have a unified policy regarding the availability of diabetes devices, resulting in significant variability among nations. Finally, technology evolves, and the list of DIY software examined in this study will eventually become outdated, as well as the devices available to patients in Norway and other international users.

## Conclusion

There is an ongoing discussion in the EU about mandatory interoperability requirements with EHR systems.^71^ Our study, set within the context of diabetes management in the EU, underscores the delicate balance between data privacy, manufacturer transparency, and individual empowerment. The challenge lies in reusing health data and accessing real-time information in a complex landscape. While real-time data access is currently unregulated with limited publicly documented APIs, developing and implementing necessary policies can potentially enhance diabetes treatment and research.

Do-It-Yourself alternatives may not align with ToS agreements, raising questions about market regulation, especially in contrast to governmental regulations. The EU has recently implemented the MDR,^72^ which covers devices used for medical purposes. Given that the diabetes devices discussed in this article fall under the MDR, one approach for promoting data access in health care is mandating publicly documented APIs and an interoperability model as proposed within the new medical device regulation.

Our call to action is for regulatory frameworks to facilitate secure and standardized health data access. The necessary technology is available; the challenge lies in interoperability and data accessibility, as outlined in our study.
